# The effect of physical exercise during radiotherapy on physical performance in patients with head and neck cancer: a trial within cohorts study protocol, the vital study

**DOI:** 10.1186/s12885-024-12172-2

**Published:** 2024-04-01

**Authors:** Yvette Kriellaars, Jorine Ariane Vermaire, Maaike Beugeling, Robert Poorter, Janneke Drijvers, Caroline Margina Speksnijder

**Affiliations:** 1 Department of Radiation Oncology, Instituut Verbeeten, Tilburg, the Netherlands; 2 Medifit Fysiotherapie Instituut Verbeeten, Tilburg, the Netherlands; 3 Department of Head and Neck Surgical Oncology, University Medical Center Utrecht, Utrecht University, Utrecht, the Netherlands; 4 Department of Oral and Maxillofacial Surgery and Special Dental Care, University Medical Center Utrecht, G05.122, 3508 GA Utrecht, P.O. Box 85.500, The Netherlands

**Keywords:** Head and neck cancer, Physical exercise intervention, Physical performance, Chemo- or bioradiotherapy, Trial within cohorts, TwiCs

## Abstract

**Background:**

During the last decade, twelve studies have been published investigating physical exercise interventions (PEIs) in patients with head and neck cancer (HNC) during radiotherapy (RT), chemoradiation (CRT) or bioradiation (BRT). These studies showed that these PEIs are safe and feasible. However, only two of these studies were randomised clinical trials (RCTs) with a satisfying sample size. Thereby, there is no cost-effectiveness study related to a PEI during RT, CRT or BRT ((C/B)RT) for patients with HNC. Therefore, the aim of this study is to investigate and compare physical performance, muscle strength, fatigue, quality of life (QoL), body mass index (BMI), nutritional status, physical activity, treatment tolerability, and health care related costs in patients with HNC with and without a 10 week PEI during (C/B)RT.

**Methods:**

This study, based on a trial within cohorts (TwiCs) design, will contain a prospective cohort of at least 112 patients. Fifty-six patients will randomly be invited for an experimental 10 week PEI. This PEI consists of both resistance and endurance exercises to optimize physical performance, muscle strength, fatigue, QoL, BMI, nutritional status, physical activity, and treatment tolerability of (C/B)RT. Measurements are at baseline, after 12 weeks, 6 months, and at 12 months. Statistical analyses will be performed for intention-to-treat and instrumental variable analysis.

**Discussion:**

This study seeks to investigate physical, QoL, and economic implications of a PEI. With a substantial sample size, this study attempts to strengthen and expand knowledge in HNC care upon PEI during (C/B)RT. In conclusion, this study is dedicated to provide additional evidence for PEI in patients with HNC during (C/B)RT.

**Trial registration:**

protocol was registered at clinicaltrials.gov with number NCT05988060 on 3 August 2023.

## Background

In 2021, the incidence of head and neck cancer (HNC) in the Netherlands was 3,174 and the prevalence was 10,635 [1]. HNC includes tumours located in four anatomical sites: pharynx, larynx, oral cavity, and sinonasal cavity [2]. The most prevalent causes of HNC are heavy consumption of tobacco and/or alcohol, and the human papillomavirus (HPV) [2].

Treatment of HNC by radiotherapy (RT), chemoradiation (CRT) or bioradiation (BRT) can cause early side effects like mucositis, odynophagia, dysphagia, xerostomia, orofacial pain, laryngeal radio necrosis, dermatitis, hair loss, nausea, and vomiting [3]. Those early side effects can cause inadequate nutrition and hydration, which can lead to loss of muscle mass, loss of muscle strength, increased fatigue, decreased physical performance, and a decreased quality of life (QoL) [4, 5]. Physical exercise interventions (PEIs) primarily focusing on resistance training have proven effective in enhancing various health-related, physiological, and disease specific outcomes like early side effects [5–7]. However, the existing evidence suggests that resistance training alone may not suffice. Therefore, incorporating endurance training into the regimen appears to be beneficial [7]. Endurance training can reduce fatigue, improve QoL, and improve physical function [7]. For an optimal effect of both resistance and endurance exercises, it is necessary to train on a moderate to hard load and intensity [8]. Thereby, it is of importance to set the PEI volume on the patient’s physical performance at the start to be able to adapt the exercise intensity depending on progress, deterioration, and symptom burden during the PEI.

During the last decade, 12 studies have been published investigating PEIs in patients with HNC during RT, CRT or BRT ((C/B)RT) treatment [9–20]. In ten studies [9–11,13–17,19,20], the PEI consisted of resistance exercises only, and in two studies [12, 18] both resistance and endurance exercises were part of the PEI. Two of these 12 studies were randomised clinical trials (RCTs) with a satisfying sample size [11, 18]. Hu et al. [11] included 146 patients in China. They showed shortly after CRT that a 60 min supervised resistance training twice a week during CRT decreased fatigue and improved QoL significantly [11]. Samuel et al. [18] included 148 patients in India and combined resistance exercises with endurance exercises in a 7 week supervised training five times a week followed by a 4 week home based training during CRT. In this study they showed a significant improvement on physical performance, measured by the six minutes walking test (6MWT), a fatigue decline and QoL improvement shortly after CRT.

To our knowledge, there is no cost-effectiveness analysis of a PEI for patients with HNC receiving (C/B)RT, while hospitals and insurances need this information due to the increasing financial burden of cancer care [21]. So it is of importance to investigate both the effects and cost-effectiveness of an exercise intervention in patients with HNC in a RCT. On the other hand, the challenge of recruitment often arises in RCTs. A good alternative to a RCT is the trial within cohort (TwiCs) design [22]. This is a design for pragmatic trials, which embeds a trial within a cohort [23]. This design can help to prevent unwanted recruitment problems due to a staged-informed consent (IC) [22]. Using this design, information about the intervention is provided exclusively after randomisation to the intervention group, decreasing the risk of disappointment bias or cross-over influences [22].

To measure the effect of a PEI, physical performance is an important outcome, as it is closely associated with QoL in patients with HNC [23]. In cancer rehabilitation, physical performance is frequently measured with parameters such as strength and walking ability [24]. Thereby, it has been shown that lower extremity muscle strength, as measured with the 30 s chair stand test (30CST), is associated with walking performance as measured with the 6MWT in older adults [25]. Both the 30SCT and 6MWT are commonly used and reliable measurement instruments to evaluate physical performance in patients with cancer [9]. Based on the fact that Samuel et al. [18], who used an optimal sample sized RCT with a PEI combining resistance and endurance training, used the 6MWT to objectively measure physical performance, the 6MWT was used as primary outcome in this study.

The aim of this study is to investigate and compare physical performance, muscle strength, fatigue, QoL, body mass index (BMI), nutritional status, physical activity, treatment tolerability of (C/B)RT, and healthcare related costs in patients with HNC with and without a 10 week PEI during (C/B)RT. Our hypothesis presumes that patients with HNC receiving the PEI will have less reduction in physical performance after (C/B)RT, as measured with the 6MWT, in comparison to patients not receiving this PEI.

## Methods

### Study design

A RCT is a powerful design for evaluating clinical interventions and is considered to generate a high level of evidence [26]. However, a RCT design has several limitations: patients may dislike the randomisation and therefore refuse participation. When patients are randomised for the control group but have a preference for the experimental intervention, drop-outs may occur. Those limitations have consequences for the recruitment and power of the study. To improve recruitment, in this study a TwiCs design will be used. The TwiCs design consists of an experimental intervention study within an observational longitudinal cohort. This design uses a staged-IC. Patients will be asked to participate in an observational longitudinal cohort study and will be informed about the design with the probability to be randomly invited for an experimental intervention which they can accept or refuse. After cohort enrolment, all patients will be randomised. This approach is feasible and efficient [27]. Patients who accept the experimental intervention will receive a 10 week PEI during (C/B)RT treatment.

### Participants

At least 112 patients will be recruited at Instituut Verbeeten, Tilburg, the Netherlands. This hospital is specialized in treating patients with HNC of which approximately 100 patients are treated with (C/B)RT yearly. Patients with HNC scheduled for (C/B)RT will be recruited by the radiation oncologist. First, patients will receive a letter explaining the study aims and procedures. Second, patients who have expressed their interest will be contacted by the local investigator and procedures will be explained in more detail. When a patient agrees, a baseline visit will be scheduled to obtain written IC and perform baseline evaluation measurements.

Patients treated with RT receive 30 times 2,2/2,1gray (Gy). Patients treated with CRT or BRT will receive seven times Cisplatin or Nivolumab combined with 35 times 2 Gy RT. This treatment will be adapted when necessary. All patients with HNC receive advices based on the ‘Dutch physical activity guideline’ [28]. Just before and during (C/B)RT, nutritional care is provided by a dietician on a weekly basis, focusing on optimal energy balance and prevention of negative energy balance.

### Inclusion and exclusion criteria

In order to be eligible to participate in this study, a patient must meet the following inclusion criteria: 1) patients with HNC who are scheduled for (C/B)RT, 2) ≥ 18 years of age, 3) sufficient Dutch writing and reading skills, 4) a Karnofsky performance status (KPS) > 60 [29], 5) able to walk ≥ 60 m without a mobility aid, and 6) no contraindication for physical activity as measured with the physical activity readiness questionnaire (PAR-Q) [30]. Patients who meet any of the following exclusion criteria will be excluded from participation in this study: (1) recurrence of HNC and/or (2) secondary HNC.

### Sample size

The sample size calculation is based on the primary outcome, the 6MWT. The power of the study is set on 80% (ß), using differences between two independent means with a 0.05 two-sided significance level. For calculation of the sample size, G*Power 3.1.9.2. was used [31]. Means and standard deviations (SDs) of the 6MWT of the study of Samuel et al. [19] were used. The first mean (SD) [483.16 (88.24)] is of patients with HNC who received the exercise intervention during (C/B)RT and the second mean (SD) [374.52 (110.26)] is of patients with HNC who received usual exercise care during (C/B)RT. Based on this, an effect size of 1.09 was calculated, which resulted in a sample size of 15 patients needed to undergo the PEI. We expect a drop-out of 24% as stated in the study of Samuel et al. [19]. Therefore, 20 patients need to be included in the PEI group. A feasibility study for PEI for patients with HNC in a Dutch population showed that 36% of the approached patients signed IC [13]. To include enough patients, a bigger sample size is needed. Using the expected participation percentage of 36%, a sample size of 56 is needed for the PEI group. Randomisation will be done in a 1:1 ratio so also 56 patients are needed in the control group. In total, 112 patients are needed. With the TwiCs design, two subgroups will arise in the PEI group, consisting of patients who accept and patients who refuse to undergo the PEI [26]. This type of non-compliance (refusal of the assigned treatment) will not be identical between the study arms [32]. Therefore, the sample size calculation will be revised when the real acceptance rate of the PEI differs from the initially estimated acceptance rate, before the end of the study [33].

### Randomisation and blinding

**Fig. 1:**
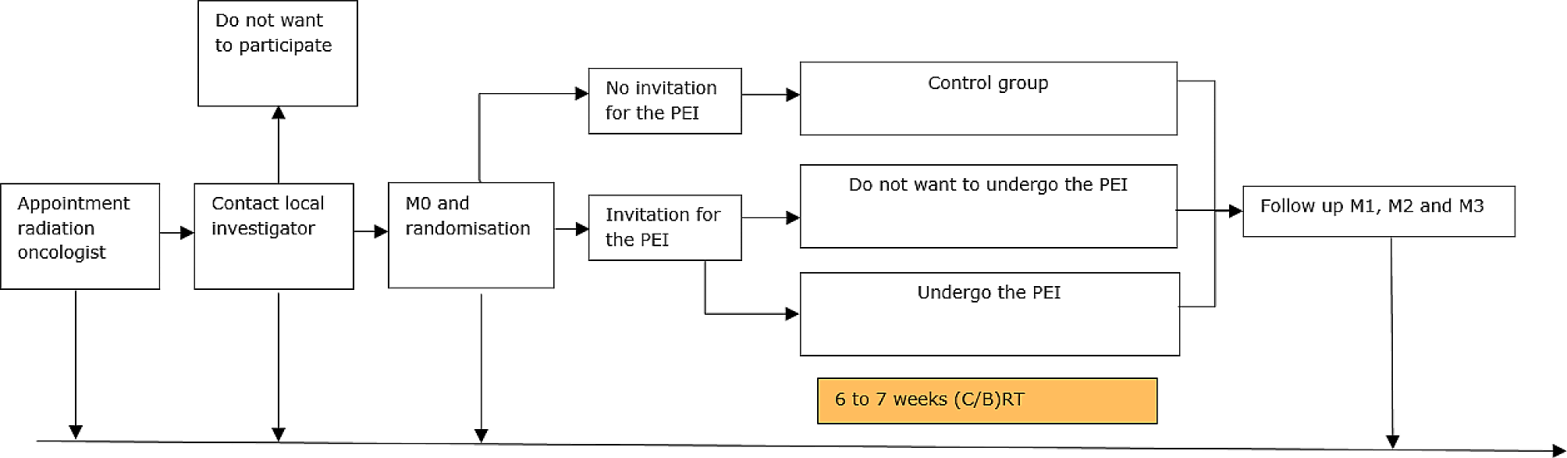
Flowchart of the recruitment of patients *(C/B)RT* chemo or bioradiation, *M0* measurement before (C/B)RT, *M1* measurement after 12 weeks, *M2* measurement after 6 months, *M3* measurement after 12 months, *PEI* physical exercise intervention

At the first appointment the radiation oncologist will provide the study information. In Fig. 1, an overview is provided of the recruitment of patients. After enough time (until one week before the start of (C/B)RT) to consider their decision, the local investigator contacts the patient and asks to participate in the cohort. When the patient agrees, a baseline measurement (M0) will be planned. At the start of this measurement appointment, the patient will sign the IC for participating in the observational longitudinal cohort study. Immediately after the baseline measurement, the data collector will randomise the patient to participate in the experimental PEI or stay in the observational longitudinal cohort. Patients who refuse to participate will be listed and asked for their reason to refuse. When a randomised patient agrees to participate in the PEI, the first appointment for the PEI will be planned. During the first PEI appointment, the IC for PEI will be signed. For randomisation, a box with 112 sealed envelopes will be used. Fifty-six envelops contain ‘invite to undergo the experimental exercise intervention’ and 56 envelopes contain ‘no invitation to undergo the experimental exercise intervention’. This study will continue until 56 patients accepted the experimental PEI. The local investigator (YK) will perform the group allocation and data collection, so this researcher will not be blinded. The physiotherapists who will provide the intervention and the patients who undergo the experimental PEI will also not be blinded.

#### Physical exercise intervention

All patients will receive usual physical exercise care. Usual care contains advise to exercise based on the ‘Dutch physical activity guideline’ [28]. This guideline advises adults to do 150 min of moderate intensive movement a week, and twice a week muscle and bone strengthening exercises. Patients who are randomised in the PEI group and accept the invitation to undergo the experimental intervention, receive a PEI for 10 weeks. The PEI will be executed 6 to 7 weeks during and 3 to 4 weeks after (C/B)RT. The PEI will be given at Medifit Fysiotherapie Instituut Verbeeten. The PEI consists of a 60 min intervention twice a week and will be supervised by a physiotherapist specialized in oncology. The PEI consists of both endurance exercises and resistance exercises. This PEI is based on two previous studies [13, 18]. During every intervention, the physiotherapist will register which endurance and resistance exercise is performed and how many sets, repetitions and which resistance per exercise is used.

Every exercise intervention starts and stops with a 10 min endurance exercise using the home trainer or treadmill. To preserve the exercise stimulus of the endurance exercises, patients will be asked to rate the endurance exercises on the 6 to 20 Borg rating of perceived exertion (RPE) [34]. A moderate to hard intensity level of these exercises are aimed, so a score between 12 and 16 RPE has to be achieved. The intensity of endurance exercise may progress over time, but may also be reduced as necessary according to the patient’s symptom burden.

Resistance exercises for the lower and upper extremity will consist of: calf raises, leg presses, lunges, squats, seated rows, lateral pull downs, bicep curls, and triceps extensions. At every PEI appointment, all resistance exercises will be executed. Before starting the PEI, muscle power is measured by a twelve repetition maximum (12RM). At the start of this program, patients will start with their 12RM score and perform 2 sets of 12 repetitions. Efforts will be made to have the exercise to rest ratio on 1 to 10, based on the duration of work period [5]. When the exercises become too easy, resistance can be increased by 5 to 10% of the 12RM. On the other hand, if it is too difficult for a patient to maintain their 12RM, for example due to symptom burden, it is acceptable to do exercises at a lower base of 5 to 10% of the 12RM.

Home-based endurance and resistance exercises three times a week is also part of the PEI. Instructions and an exercise diary will be provided by the physiotherapist at the first appointment. The patients will be asked to fill in this diary after every exercise session at home. This diary will be discussed weekly with the patient by the physiotherapist to repeat instructions and encourage the patient to continue exercising at home as much as needed. Endurance exercise contains a 30 min walking schedule. Resistance exercises are based on the exercises patients can perform without instruments: calf raises, squats, lunges and wall sits for lower extremity, and push-ups against the wall, side raises and front raises for upper extremity. Patients start with 2 sets of 12 repetitions. Self-efficacy has shown to have an impact on health practices and adaptation to cancer and cancer treatment [35]. By combining supervised exercising with individual home based exercises we aim to increase self-efficacy.

### Measurements

The socio-demographic data (age, gender, education, employment and marital status) and medical data (tumour site, disease stage, comorbidity, HPV status, tobacco and alcohol use, nutrition status and type of treatment) will be assessed at baseline (M0). Physical performance, muscle strength, fatigue, QoL, BMI, nutritional status, and physical activity will be assessed at baseline (M0), 12 weeks (M1) and 6 (M2) and 12 (M3) months after baseline. Treatment tolerability will be assessed at M1, M2 and M3. Health related costs will be collected at M3. All tests and Dutch questionnaires are validated and have satisfying clinimetric properties.

### Primary outcome

Physical performance will be measured by the 6MWT [36]. The 6MWT measures the walking distance in meters of a patient in 6 min on a 10 m parkour.

### Secondary outcomes

Muscle strength will be assessed by grip strength [37], lower and upper body strength and the 30 s chair stand test (30SCST) [38] according the standard procedures of these tests. We developed a measurement protocol how to perform the measurements. For the grip strength [kg], the JAMAR^®^ grip strength dynamometer will be used [37]. To assess lower (knee flexion and extension) and upper body strength [N] (elbow flexion and extension), the microFET^®^ hand held dynamometer will be used according to standardized procedures by 3 times testing [39]. The 30SCST will measure the number of completed stand ups on the patients’ fastest pace over a 30-second period.

Fatigue will be measured using the multidimensional fatigue inventory (MFI) which is a 20-item questionnaire assessing five dimensions: general, physical and mental fatigue, and reduced activity and motivation [40].

The following questionnaires will be used to measure QoL: the European organisation for research and treatment for cancer quality of life questionnaire (EORTC QLQ-C30), the EORTC QLQ head and neck module (EORTC QLQ-H&N43), and the EuroQol- five dimensions- five level (EQ-5D-5 L). The EORTC questionnaires are designed to be cancer-specific, multidimensional in structure, appropriate for self-administration and applicable across a range of cultural settings. The EQ-5D-5 L is a standardized instrument which can be used as a quantitative measure of health outcome and reflects the patient’s own judgement. Dutch reference values are available [41–43].

Height and weight in light clothing will be measured, from which body mass index (BMI) in kg/m^2^ will be calculated [44].

The short nutritional assessment questionnaire (SNAQ) consists of three questions and will be used to monitor nutritional status and assess the risk of malnutrition [45].

Physical activity will be measured by the short questionnaire to assess health enhancing physical activity (SQUASH), including commuting activities, leisure time activities, household activities, and activities at work and/or school [46].

(C/B)RT tolerability will be retrieved from medical records and registered as the percentage of scheduled treatment completion, percentage of (C/B)RT adjustment and type of (C/B)RT adjustment. Furthermore, toxicity will be registered from medical records, which includes the presence of pain, mucositis, dysphagia, aspiration, nausea and vomiting, and dry mouth according to the common terminology criteria for adverse events v 4.0 (CTCAE). To assess health related costs, a cost-effectiveness analysis will be performed for one year. Treatment data will be retrieved from medical records. Price levels of the years during this study will be used. By using the EQ-5D-5L and health related costs, the quality-adjusted life years (QALYs) can be calculated.

### Statistical analyses

To test normality of continuous data, the Shapiro-Wilk test will be used. Continuous data will be presented descriptively with means and SDs. When continuous data are not normally distributed, the median and inter quartile range (IQR) will be presented. For ordinal data, both the median and IQR will be presented. Nominal data will be presented as numbers and percentages. Comparison between normally distributed groups will be done with the independent t-test when data are normally distributed. For non-normally distributed data, the Mann-Whitney U test will be used. Nominal data will be compared by the Chi-square test or Fisher-exact test when a particular cell is < 5. For all different groups, statistical analyses will be performed for intention-to-treat (ITT) and instrumental variable analysis [47]. An interim analysis will be conducted after 26 patients underwent the PEI to evaluate the power calculation. The incremental cost-effectiveness ratio (ICER) will be calculated as the difference in costs divided by the difference in QALYs between groups. When data are missing, ‘available case analyses’ will be used, indicating that missing cases will be discarded in the variables that are needed for a specific analysis. All analyses will be performed using statistical package for the social sciences (SPSS). A *p*-value below 0.05 is considered statistically significant.

## Discussion

HNC itself, but also side effects of (C/B)RT sush as inadequate nutrition, weight loss, loss of muscle mass, loss of muscle strength, fatigue, and QoL can be deteriorated by a PEI [4]. Therefore, the aim of this study is to investigate and compare physical performance, muscle strength, fatigue, QoL, BMI, nutritional status, physical activity, treatment tolerability of (C/B)RT, and healthcare related costs in patients with HNC with and without a 10 week PEI during (C/B)RT. It has been demonstrated that PEI in patients with HNC during (C/B)RT is feasible and safe, reducing the deterioration of physical performance and QoL during (C/B)RT [9–20].

This study presents various practical and operational challenges which can involve the performance of this study. Blinding of the local investigator responsible for follow-up data collection is not feasible due to the small scale of the hospital, making it apparent which patient receives the PEI and which patient does not. Consequently, the local investigator performs both randomisation and data collection, a decision aimed at enhancing participant comfort and convenience by limiting interactions to one investigator.

With the intention to prevent a potential sample bias, a diverse and inclusive cohort of patients with HNC will be selected [48]. Consequently, the population participating in this study will be subjected to a varied spectrum of treatments within (C/B)RT. This comprehensive selection is made in the pursuit of enhancing the generalizability of the findings.

In clinical practice, patients receiving (C/B)RT experience a demanding and time-consuming schedule. Their schedule includes multiple appointments with healthcare professionals, including a radiation oncologist, dietician, and dental hygienist. This high frequency of appointments may lead to refusal of study participation [12]. In an effort to accommodate and facilitate participation, we prepared a strategy to schedule study appointments just before or after a patient’s usual care appointment at Instituut Verbeeten.

This study marks the first use of a TwiCs design to investigate the effect of a PEI in patients with HNC during (C/B)RT. We have chosen this innovative approach for its potential advantages in recruitment rates. This design reflects the clinical practice using a staged-informed consent. Nonetheless, it is worth noting that effect estimation may be more challenging when compared to a standard RCT, because the ITT approach slightly differs from the ITT definition in a RCT [49].

Crucially, this study represents the first attempt to investigate the cost-effectiveness of a PEI for patients with HNC. Giving the increasing number of people surviving from cancer, cancer care needs to be kept affordable. Economic evaluation of interventions play a pivotal role in decision-making in the context of delivering affordable cancer care in high-income countries [21].

This study seeks to investigate physical performance, muscle strength, fatigue, QoL, BMI, nutritional status, physical activity, treatment tolerability of (C/B)RT, and healthcare related costs of this PEI. With a substantial sample size this study attempts to strengthen and expand upon the conclusion of previous studies. It Addresses the existing research gap concerning a PEI in patients with HNC by aiming to decrease the decline of physical performance and QoL during (C/B)RT and gaining insight in the healthcare related costs. In conclusion, this study is dedicated to provide additional evidence in physical cancer care in patients with HNC during their (C/B)RT.

## Data Availability

Not applicable.

## Abbreviations

BRT: Bioradiation
(C/B)RT: Chemo- or bioradiotherapy
CRT: Chemoradiation
CTCAE: Common Terminology Criteria for Adverse Events v 4.0
EORTC QLQ-C30: European Organisation for Research and Treatment for Cancer Quality of Life Questionnaire
EORTC QLQ-HN43: European Organisation for Research and Treatment for Cancer Quality of Life Head and Neck Module
EQ-5D-5 L: EuroQol- Five Dimensions- Five Level
ETZ: Elizabeth Tweesteden Ziekenhuis
Gy: Gray
HNC: Head and Neck Cancer
HPV: Human Papilloma Virus
IC: Informed Consent
ICER: Incremental Cost-Effectiveness Ratio
IQR: Inter Quartile Range
ITT: Intention To Treat
KPS: Karnofsky Performance Status
MFI: Multidimensional Fatigue Inventory
NWHHT: Dutch Head and Neck Society
PAR-Q: Physical Activity Readiness Questionnaire
PEI: Physical Exercise Intervention
QALY: Quality-Adjusted Life Year
QoL: Quality Of Life
RPE: Rating of Perceived Exertion
RT: Radiotherapy
RCT: Randomised Controlled Trial
SD: Standard Deviation
SF-36: Short Form 36 questionnaire
SNAQ: Short Nutritional Assessment Questionnaire
SPSS: Statistical Package for the Social Sciences
SQUASH: Short Questionnaire to assess health enhancing physical activity
TwiCs: Trial Within Cohorts
12RM: Twelve Repitition Maximum
6MWT: Six Minute Walking Test
30SCST: Thirty Seconds Chair Stand Test
